# Census of the Blockley Alms House and Philadelphia Hospital, January 10, 1844

**Published:** 1844-03-23

**Authors:** 


### THE MEDICAL EXAMINER,

### And Record of Medical Science.

Vol. VII.] PHILADELPHIA, SATURDAY, MARCH 23, 1844. [No. 6.

### CENSUS OF THE BLOCKLEY ALMS HOUSE AND PHILADELPHIA HOSPITAL,

*January* 10, 1844.

RECAPITULATION.

| | | |
|---|---|---|
| White men, - - - | 976 | |
| " women, - - - - | 533 | |
| | | 1509 |
| " boys, - - - - | 126 | |
| " girls, - - - - | 31 | |
| | | 157 |
| Black men, - - - | 100 | |
| " women, - - - | 113 | |
| | | 213 |
| " boys, - - - - | 9 | |
| " girls, - - - - | 11 | |
| | | 20 |
| | | 1899 |

*Report of the Obstetric Department of the Philadelphia Dispensary, for the year* 1843. By JOSEPH WARRINGTON, M. D.

Number of women delivered, at or near the full period of gestation, . . . . . 179

Number of children born, { males, 109 ; females, 74 } Total, 183

A simple cell or vesicle is the very initiatory development, the *genesis* of every structure, in both the animal and vegetable kingdoms.—*Dr. Power.*

